# Different patterns of age-related central olfactory decline in men and women as quantified by olfactory fMRI

**DOI:** 10.18632/oncotarget.16977

**Published:** 2017-04-08

**Authors:** Brittany Martinez, Prasanna Karunanayaka, Jianli Wang, Michael J. Tobia, Megha Vasavada, Paul J. Eslinger, Qing X. Yang

**Affiliations:** ^1^ Department of Radiology, Center for NMR Research, The Pennsylvania State University College of Medicine, The Milton S. Hershey Medical Center, Hershey, PA, USA; ^2^ Department of Neurology, The Pennsylvania State University College of Medicine, The Milton S. Hershey Medical Center, Hershey, PA, USA; ^3^ Department of Neurosurgery, The Pennsylvania State University College of Medicine, The Milton S. Hershey Medical Center, Hershey, PA, USA

**Keywords:** neuroimaging, olfaction, fMRI, sex differences, presbyosmia

## Abstract

Age-related olfactory decline, or presbyosmia, is a prevalent condition with potentially devastating consequences on both quality of life and safety. Despite clear evidence for this decline, it is unknown whether presbyosmia is sex-dependent and also whether it is due to central or peripheral olfactory system deterioration. Therefore, the goals of this study were to investigate the neurofunctional substrate of olfactory decline and examine its relationship to age and sex in thirty-seven (18 women, 19 men) healthy older participants using olfactory functional MRI (fMRI). The olfactory fMRI paradigm utilized unique odor+visual and visual-only conditions to contrast peripheral-to-central and central-to-central olfactory processing, respectively. Age was negatively correlated with fMRI activation in olfactory-related regions. Significant aging effects were identifiable in male participants in all target regions. Female participants, however, showed a different pattern of functional decline. Extended unified structural equation modeling (euSEM) analysis revealed that the effective functional connectivity profile was drastically different between male and female participants, with females manifesting a top-down mechanism to offset age-related olfactory activation decline. Our results support the hypotheses that the central olfactory system is involved in age-related olfactory decline, and that resilience to age-related olfactory decline in women may be due to their profuse olfactory network effective connectivity.

## INTRODUCTION

Along with other sensory systems, the sense of smell declines with age in a condition known as presbyosmia, which affects over half of individuals between the ages of 65 and 80 and between 62-80% of those over the age of 80 [1, 2]. Many studies have documented the effect of normative aging on the human olfactory system, with elderly individuals frequently presenting with odor threshold, memory, and identification deficits [3-6]. Beyond normative olfactory decline, severe olfactory deficits are considered to be early symptoms for several neurodegenerative diseases, such as Alzheimer’s disease and Parkinson’s disease [7, 8]. In addition, due to the fact that “flavors” of food are predominately mediated by the sense of smell, presbyosmia has adverse effects on both quality of life and proper nutrition [2, 9]. Despite clear evidence for age-related olfactory function decline, the precise neurofunctional substrate of this biological process still remains elusive [10].

The olfactory system comprises central and peripheral olfactory systems. The peripheral olfactory system includes olfactory epithelium and nerve; while, and central olfactory nervous system includes the olfactory bulb and tract, piriform cortex, anterior olfactory nucleus, olfactory tubercle, and part of entorhinal cortex and amygdala. While the peripheral components function to detect odors, the central components process and integrate olfactory afferent signals and other sensory stimuli to form an odor percept [11, 12]. From a neuropathological perspective, the question of how this natural olfactory decline that is observed behaviorally relates to degenerations of the central and peripheral olfactory nervous system is still largely unknown [10]. Along the same lines, though several behavioral studies have demonstrated that women typically outperform men on olfactory tasks during normal aging, it is unclear whether this is due to differences in peripheral sensory function or central cognitive processing of olfactory information [4, 13]

Though there is still much to be learned, functional magnetic resonance imaging (fMRI) studies have been at the forefront of seeking the answers of these questions and have significantly contributed to the current understanding of the effects of age and sex on olfactory function. Specifically, fMRI studies have found that activation in olfactory-related structures, such as the piriform cortex, the amygdala, and the entorhinal cortex, is decreased in elderly individuals compared to young, healthy subjects [14-16]. Additionally, for the middle age range, a recent fMRI study on sniffing behavior found significant age- and sex-related decline in second-order olfactory structures, with men displaying significant aging effects [17]. On a similar note, decreased activation in olfactory-related regions of the cerebellum has also been observed in elderly subjects [18]. Electrophysiological studies have reported significantly longer latency and weaker amplitude of olfactory event related potentials in older adults [19, 20]. In summary, these studies provide evidence for the effects of aging and sex on olfactory function and have also highlighted the utility of neuroimaging techniques in evaluating these relationships. However, as previously mentioned, the specific neural correlates underlying the prominent behaviorally-observed sex differences in olfactory performance remains largely undefined.

Therefore, the goal of this study was two-fold. First, this study sought to evaluate the effect of age on neural activity in the central olfactory system in older, cognitively normal participants using an olfactory-visual association fMRI paradigm that was specifically designed to investigate central olfactory system function [21]. Based on previous findings, it was hypothesized that a significant effect of age on central olfactory neural activity would be observed. Secondly, this study sought to uncover the neural correlates of the sex differences in aging of the central olfactory system. Based on previous behavioral studies, it was hypothesized that men would display significantly greater deficits in central olfactory system activation compared to women.

## RESULTS

Based on the neuropsychological evaluations, all male and female participants were within the range of healthy, normal cognition and no sex differences were observed in the results of Mini-Mental State Examination (MMSE) (males = 28.47 ± 1.39, females = 28.06 ± 1.86 Mattis Dementia Rating Scale-2 (DRS-2) (males = 13.00 ± 1.76, females = 12.89 ± 1.57), or CVLT-II (males = 63.11 ± 14.99, females = 62.06 ± 11.35).

**Figure 1:**
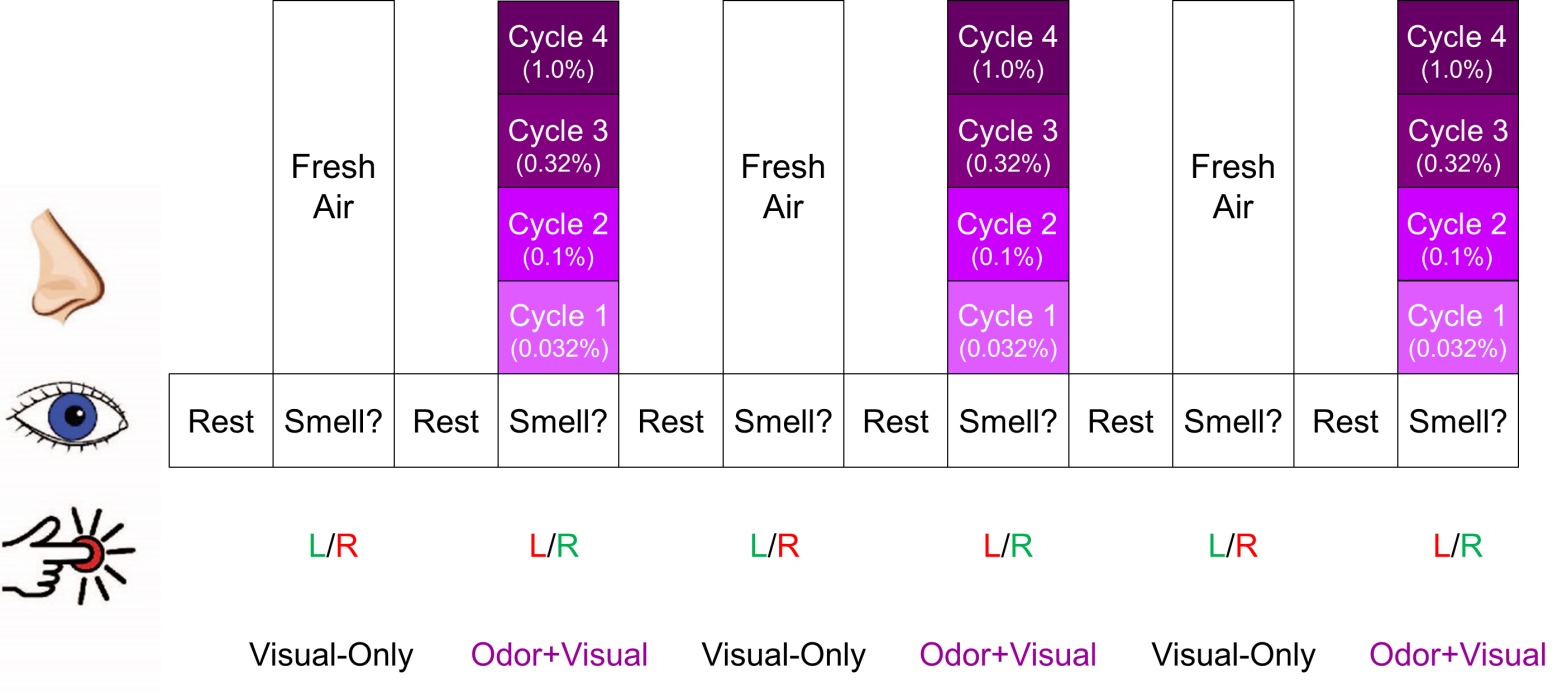
Olfactory fMRI paradigm Each intensity of lavender was presented 3 times. Every time the visual cue “Smell?” appeared on the screen, the participant was instructed to respond with a right button press if they smelled lavender odor and a left button press if they did not. Green lettering indicates the correct response for each condition, whereas red indicates incorrect response. The cycle shown was repeated 4 times.

**Figure 2:**
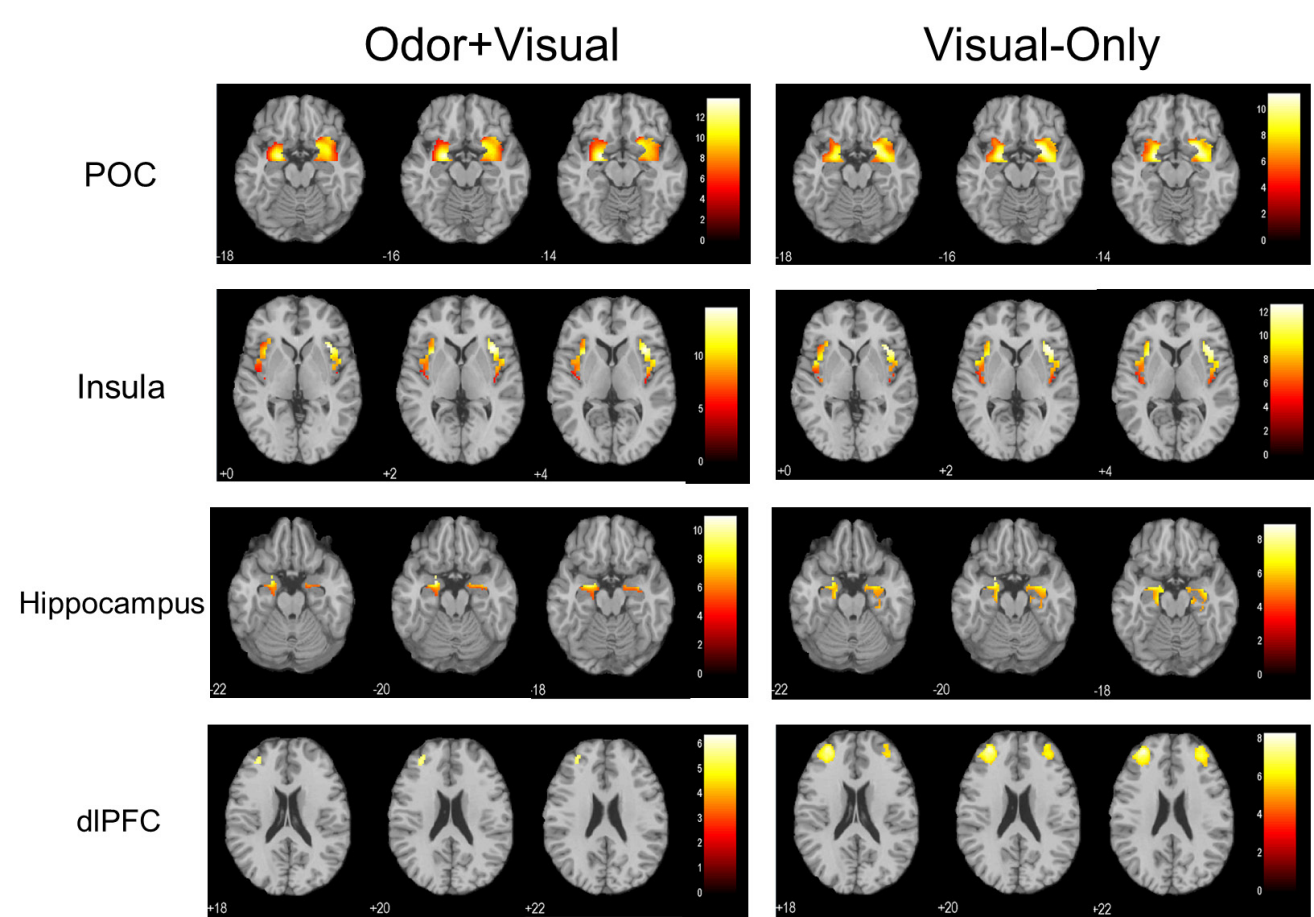
One sample t-tests showing significant activation in the primary olfactory cortex (POC), insula, hippocampus, and dorsolateral prefrontal cortex (dlPFC) (*p* < 0.05, Family-wise error (FWE) corrected, extent threshold = 10) during the olfactory paradigm for both odor+visual and visual-only conditions

Table 1 shows the activated regions for both the odor+visual and visual-only conditions using the olfactory fMRI paradigm in Figure 1. Significant activation (*p* < 0.05, Family-wise error (FWE) corrected) for both conditions was observed in the primary olfactory cortex (POC), insula, hippocampus, and dorsolateral prefrontal cortex (dlPFC) as shown in Figure 2. The linear regression analysis (*p* < 0.001, uncorrected) detected significant negative correlation between age and the activation in the POC, insula, and dlPFC for both the odor+visual and visual-only conditions (Table 2). The slopes of the age curves for the odor+visual and visual-only conditions were not statistically different in any of the predefined regions of interest (ROI).

**Table 1:** Peak activation for olfactory regions during odor+visual and visual-only conditions.

Odor+Visual	Region	Size	Coordinates			*T*	*P*_FWE_
			**X**	**Y**	**Z**		
	POC	988	-24	-2	-14	13.86	<0.001
	Insula	795	34	18	0	14.67	<0.001
	Hippocampus	504	-20	-4	-16	11.20	<0.001
	dlPFC	456	6	42	44	6.36	<0.001

Visual-Only	Region	Size	Coordinates			*T*	*P*_FWE_
			**X**	**Y**	**Z**		
	POC	1037	-24	-2	-12	11.23	<0.001
	Insula	808	34	20	2	12.66	<0.001
	Hippocampus	649	-18	-6	-14	8.92	<0.001
	dlPFC	772	-32	50	24	8.29	<0.001

Notes: One-sample t test

Family-wise error (FWE) corrected, p < 0.05, cluster size > 10 voxels.

POC = Primary Olfactory Cortex, dlPFC = Dorsolateral Prefrontal Cortex

**Table 2:** Negative age correlations for both odor+visual and visual-only conditions.

	Odor+Visual		Visual-Only	
	r	*p*	r	*p*
POC	0.465	0.0043	0.486	0.0026
Insula	0.602	0.0001	0.543	0.006
Hippocampus	0.374	0.0248	0.342	0.0413
dlPFC	0.449	0.0053	0.53	0.0007

Notes: POC = Primary Olfactory Cortex, dlPFC = Dorsolateral Prefrontal Cortex

**Figure 3:**
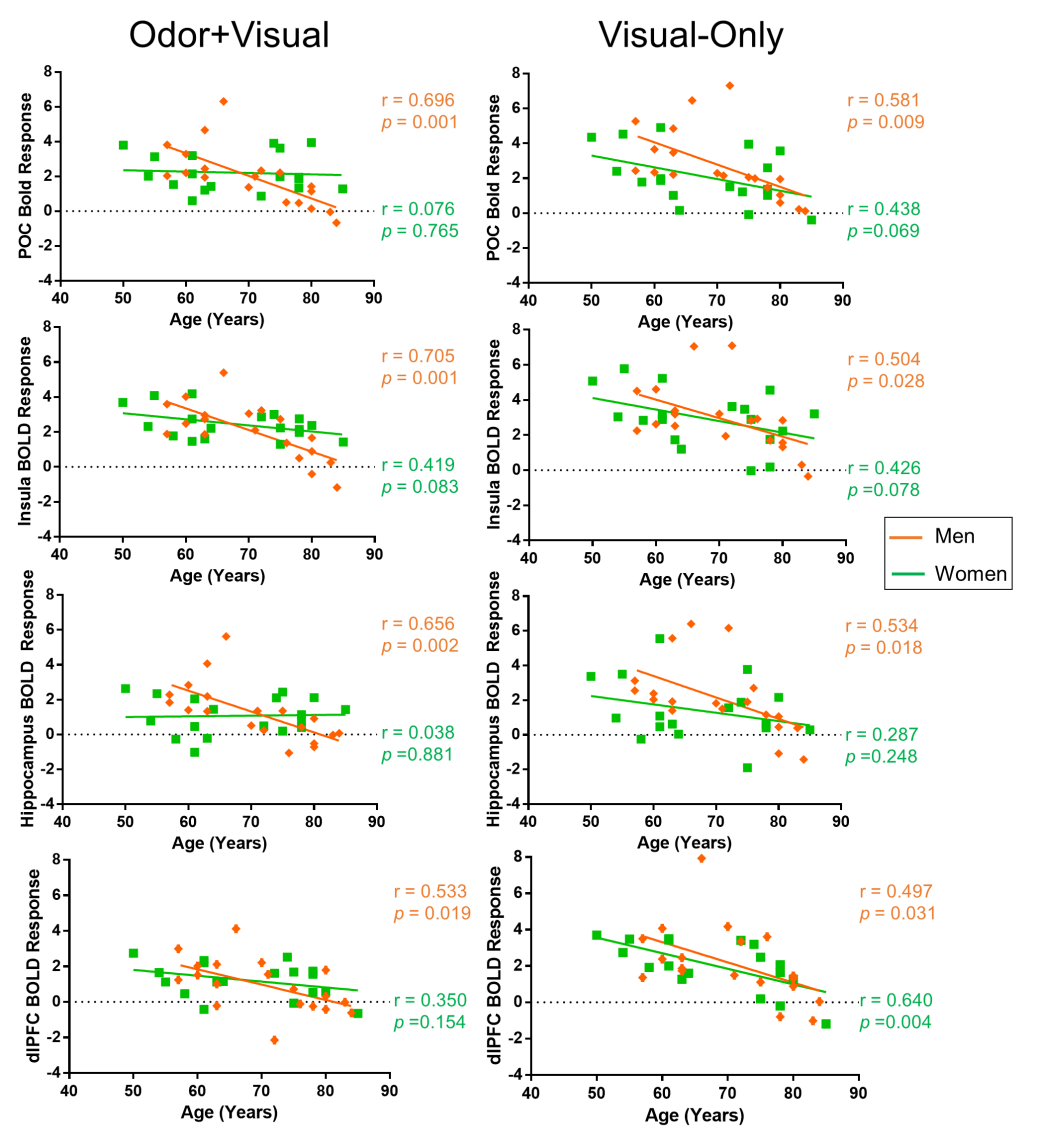
Sex differences in BOLD response Primary olfactory cortex (POC), insula, hippocampus, and dorsolateral prefrontal cortex (dlPFC) BOLD responses to odor+visual and visual-only conditions in men and women. The difference between men and women was found to be significant in the POC (*p* = 0.006), insula (*p* = 0.0164), and hippocampus (*p* = 0.0056) during the odor+visual condition. However, no significant difference was observed between the two sex groups during the odor+visual condition in the dlPFC (*p* = 0.1861) during the odor+visual condition or in the POC (*p* = 0.2811), insula (*p* = 0.4807), hippocampus (*p* = 0.2342), or dlPFC (*p* = 0.6269).

Figure 3 shows the age correlations with the BOLD signal for male and female groups during odor+visual and visual-only conditions in four brain structures. Significant differences were found between male and female groups in the slopes of the age curves during the odor+visual condition. Under this condition, the male group exhibited a highly significant age decline in BOLD signal, while the female group did not. In contrast, under visual-only condition, age-related BOLD signal declines were found in both groups. Behaviorally, there was no significant difference between the scores of the University of Pennsylvania Smell Identification Test (UPSIT) on the male and female participants (females = 33.82 ± 1.142, males = 32.94 ± 0.91) and UPSIT score was not found to be significantly correlated with age in either group. The fMRI-related behavioral responses, however, were significantly faster for female participants than for male participants (p = 0.0034) in the odor+visual condition. Additionally, UPSIT score was not significantly correlated with the BOLD response of any of the four ROIs in either condition.

**Figure 4:**
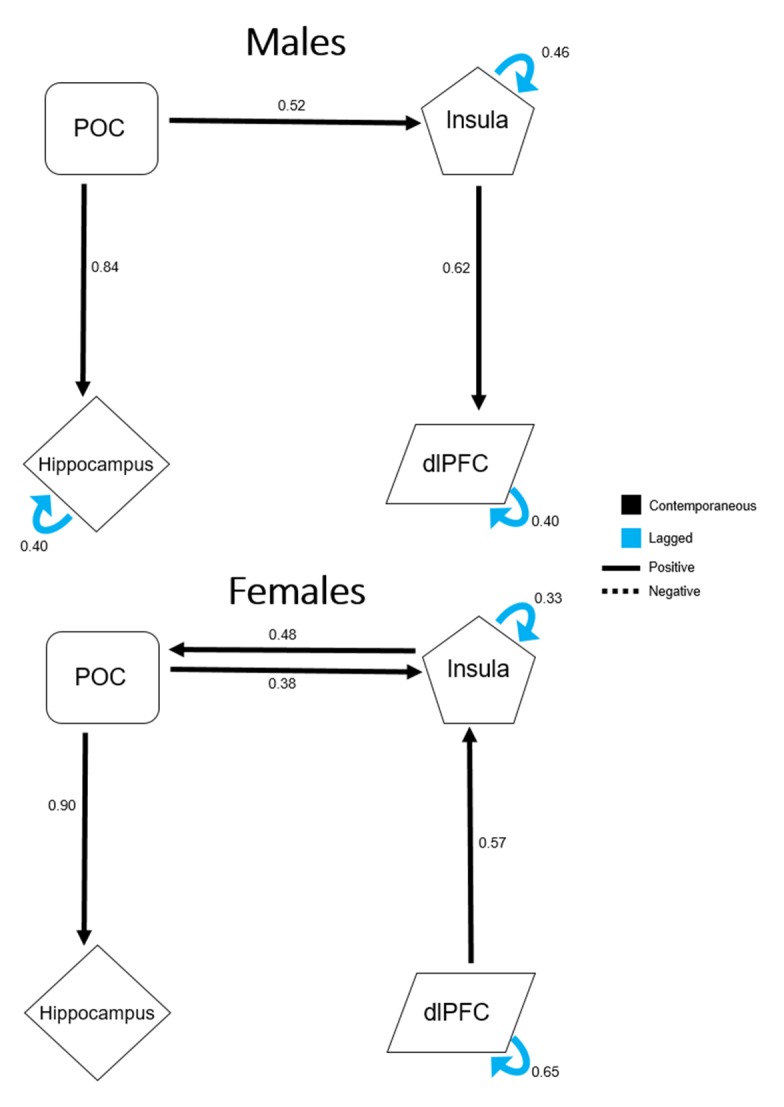
Extended unified structural equation modeling results for male and female participants Values represent the average β estimates. The presented model for male participants demonstrates output from the primary olfactory cortex (POC), but no direct input into it. The model for female participants contains direct input into the POC from secondary olfactory structures, indicating a possible compensatory mechanism to offset age-related central olfactory system decline, specifically during odorant processing.

Figure 4 shows a direct comparison of the respective extended unified structural equation modeling (euSEM) models for male and female groups estimated using time courses of the four *a priori* defined ROIs. These models revealed prominent differences in effective functional connectivity patterns between males and females during the performance of this olfactory fMRI paradigm. Specifically, the model for the male group identified mostly output connections from the POC to secondary olfactory structures, such as the insula, hippocampus, and to the dlPFC via the insula. In contrast, the model for the female group identified bidirectional connections between the POC and the insula. Furthermore, opposite directional connectivity was found between the insula and the dlPFC in females when compared to the model obtained for the male group. However, when the euSEM data for all participants were stratified according to sex, no significant differences were found between the connection strengths of the common directional connectivities of male and female groups.

## DISCUSSION

Our paradigm produced significant activation in the POC for both the odor+visual and visual-only conditions. Note that during visual-only conditions, the activation in the POC was produced by a visual cue without an odor. As demonstrated previously, participants were undergoing rapid odor-visual association during this paradigm, in which likely an association between the visual cue “Smell?” and an odor stimulus was formed, thereby eliciting a similar activation response in the POC by a subsequent visual cue [21]. This paradigm, therefore, provides an effective way to stimulate the central olfactory system even in the absence of an afferent olfactory sensory signal.

The results of this study revealed a significant effect of age on the central olfactory nervous system. Specifically, the linear regression analysis showed a significant negative correlation between age and the BOLD signal during both the odor+visual and visual-only conditions for all of the central olfactory structures. These data support the hypothesis that a significant decrease in BOLD signal in the brain in response to our olfactory paradigm reflects an age effect in the central nervous system. During the odor+visual condition, both the peripheral and central olfactory systems are actively involved in odor processing. Specifically, under this condition, lavender odorant molecules bind to the receptors of the olfactory sensory neurons (OSNs) located peripherally in the epithelium of the nasal cavity. These neurons project to the olfactory bulb by means of the olfactory nerve and subsequently to the POC, the major component of the central olfactory system, and other higher-order cortical areas via mitral and tufted cells [10, 22, 23]. The POC then sends olfactory information to secondary olfactory structures, such as the insula, hippocampus, and dlPFC, interactively. On the other hand, as demonstrated previously [21], during visual-only conditions, the central olfactory structures are also activated by the visual cues that have been paired with an odor in preceding odor+visual conditions. The activation in this case is presumably being produced without any involvement of the peripheral olfactory nervous system. Furthermore, in the same previous study, with an alternative paradigm during which no odor was presented throughout, there was no significant POC activation observed by the visual cue, suggesting that the initial pairing of the odor and visual stimulus was necessary to elicit the central olfactory activation in the subsequent visual-only conditions. As such, the significant age-related BOLD signal decline observed in this study during the visual-only condition should reflect an age effect on the central olfactory system. Thus, our odor-visual association paradigm provides an effective avenue to isolate contributions of the peripheral nervous system from that of the central system during olfactory processing.

As shown in Figure 3, the BOLD responses to odor+visual stimulations exhibited a clear trend of difference in the effect of age between female and male groups. While there was a significant decline in the BOLD signal of central olfactory structures with age in the male group, only the insula showed a trend of decline in the female group. In contrast, visual-only conditions showed trends of age-related decline in the BOLD signal in the four brain structures and for both sexes.

To better understand this apparent sex difference in the age effect from a systems level perspective, an euSEM was performed and the results in Figure 4 revealed markedly different effective functional connectivity models between males and females during this paradigm. Specifically, for the male group, there were unidirectional connections from the POC to the hippocampus and the insula, and also from the insula to the dlPFC. For the female group, however, there were bidirectional connections of the POC to and from the insula. The insula also received directed input from the dlPFC, a connection that was opposite in male participants. Although this model of effective functional connectivity was far from complete, it revealed that, compared to males, the POC of female participants, tends to receive greater input from secondary structures, a tendency for a top-down mechanism during olfactory processing. Assuming that, with an odor stimulation, the afferent signal from the peripheral sensory nerves to the POC declines with age, then the activation of the POC must also be reduced, as has been previously reported [14-16]. For women, however, the bidirectional effective connectivity between the POC and secondary olfactory structures could provide an effective compensatory mechanism that is utilized to offset age-related decline in the olfactory nervous system. This compensatory mechanism for odor processing could explain why age-related decline was more apparent in visual-only conditions. Under this condition, neuronal activity in the POC is driven by the central olfactory system. Thus, the observed age decline of the BOLD signal should predominantly reflect an effect of age on the central olfactory system where compensatory mechanisms for perceiving odors in females are no longer effective.

The differences in euSEM models between male and female groups may also highlight fundamental differences in processing olfactory sensory stimuli between men and women, which provides a plausible interpretation from a functional connectivity perspective for the observed sex differences in olfactory behavioral measurements across studies. This notion has been supported by previous chemosensory studies that have shown that women appear to allocate greater attention to intranasal stimuli compared to men, thus suggesting an implicit difference in the cognitive processing of sensory stimuli in men and women [24, 25]. Similarly, it has been previously pointed out that the sex dimorphisms of both cerebral hemispheres and asymmetry could also be a potential factor in the clear sex differences observed in chemosensory processing [26].

On a special note, dlPFC activation is typically not observed in response to odor stimulation. However, our paradigm has been shown to evoke rapid association of odor and visual cues, where working memory is involved [21]. With this paradigm, the dlPFC was activated during odor and visual pairings and during subsequent visual-only conditions. As seen in Figure 3, it is intriguing that for the visual-only condition, both male and female participants show a similar effect of age in all four brain structures, including the dlPFC. One plausible explanation for this is since the dlPFC is not directly involved in basic odor processing, but rather in higher-order cognitive processing, such as working memory, the negative correlation of activation with age in the dlPFC could reflect the effect of age on the central nervous system. Our previous study of the fMRI time-course data obtained using this paradigm from a young cohort indicated that the activation in the dlPFC during the visual-only condition temporarily leads to activation in the olfactory structures via the hippocampus, indicating that the dlPFC may be involved in triggering activations in olfactory structures by the visual cue [21].

There were several limitations to this study. First, the sample size of the cohort was relatively small. With a few more subjects of 65-70 years, the results would be significantly stronger. Second, in order to fully understand the olfactory changes occurring over the lifetime, a middle-aged population would need to be included in future studies. Future research should also focus on determining the age range where these olfactory deficits typically begin and if there are environmental, health, and/or other factors that possibly promote or protect against such changes. Finally, several lines of evidence, particularly in rodents, have indicated a deterioration in the peripheral olfactory system could be a cause of age-related olfactory decline [1, 10]. While the cohort of this study was carefully screened via questionnaires to rule out potential clinical issues in peripheral olfactory system and evaluated behaviorally with UPSIT, there was no biological data collected in the peripheral olfactory system such that the contribution of the aging effect from the peripheral system could not be evaluated concurrently.

Overall, with our novel olfactory fMRI paradigm, we demonstrated a clear age-related activation decline in the central olfactory system in our cohort. In addition, we observed sex differences in the relationship of olfactory activation with age. Our euSEM analysis suggested that such sex difference in aging characteristics could be attributed to the differences in odor perception between men and women. Our data and analyses supported the hypothesis that the central olfactory system is involved in age-related decline that is observed behaviorally in olfactory performance. Furthermore, the results provide normative aging data that are essential for generating a more comprehensive profile of age-related olfactory decline in both men and women. Taken together, these findings highlight the central olfactory system as a source for presbyosmia and that prominent sex differences in age-related olfactory behavior could be attributed to the differences in causal functional connections of central olfactory structures during odor perception.

## MATERIALS AND METHODS

### Participants

Older (n=37, mean age = 69.2 ± 9.6 years, 18 women, 19 men), cognitively healthy participants (age range = 50-85 years) were recruited from the community through advertisement for the study. Prior to study participation, participants were screened for conditions related to olfactory dysfunction (e.g. allergies, head trauma, viral infections), psychiatric and neurological conditions, and MRI safety (e.g. metal implants, claustrophobia). The study protocol was approved by the Pennsylvania State University College of Medicine Institutional Review Board, which required that each subject give verbal and written informed consent before participating.

### Behavioral tests

Olfactory function of all participants was evaluated using the UPSIT, (Sensonics Inc., Haddon Heights, NJ, USA). The UPSIT is a self-administered, forced-choice, scratch-and-sniff standardized test, consisting of 4 booklets containing 10 odorants each, with scores ranging from 0-40. [4, 27]. UPSIT scores were analyzed for the effects of age and sex and were also used in correlative analyses of the fMRI data.

Normal cognition and learning ability was assessed using three neuropsychological evaluations, including the MMSE, DRS-2, and CVLT-II. The MMSE is a brief, 11-question screen that provides a measure of cognitive status in adults by testing five areas of cognition, including registration, attention, recall, orientation, calculation, and language [28]. The CVLT-II is a comprehensive and detailed assessment of verbal learning and memory for older adolescents and adults that consists of five learning trials of 16 words [29]. CVLT-II scores are reported in this study as sex- and age-scaled short-term memory scores (*T* score). Finally, the DRS-2 is composed of 36 tasks and 32 stimulus cards that aim to evaluate an overall level of cognitive functioning, with five subscales that provide further information on attention, construction, conceptualization, initiation/perseveration, and memory [30]. DRS-2 data reported for this study represent the age-corrected scaled scores for the total test score.

### Olfactory fMRI paradigm

The olfactory fMRI paradigm is shown in Figure 1, consisting of alternating “odor+visual” and “visual-only” conditions. During odor+visual conditions, an odorant was presented for a duration of 6 S simultaneously with the visual cue “Smell?” and then followed by 12 S of fresh air with the visual cue “Rest.” During visual-only conditions, the same visual cue “Smell?” was presented for 6 S simultaneously with fresh air. Lavender oil was used as the stimulation odorant (Givaudan Flavors Corporation, East Hanover, NJ, USA) because it is typically perceived as pleasant in the general population and has minimal trigeminal stimulation [31-33]. The odorant was delivered bilaterally to each participant’s nostrils directly using an MR-compatible olfactometer (Emerging Tech Trans, LLC, Hershey, PA, USA) at a constant airflow rate of 6 L/min at room temperature and 50% relative humidity. Four intensities of lavender (0.032%, 0.10%, 0.32%, and 1.0% concentrations diluted in 1,2-propanediol (Sigma, St. Louis, MO, USA)) were administered sequentially. This protocol consisting of incremental odor intensities was previously shown to effectively minimize the habituation effects on BOLD signal [31]. Each odor intensity was presented 3 times before being increased to the next intensity. The cycle was repeated 4 times and the odor intensities were presented from weakest to strongest.

To ensure task compliance, each time the visual cue “Smell?” appeared on the screen, participants were asked to respond with a button press corresponding to whether or not they smelled the odor. Participants were instructed that a left hand button press indicated “no” and a right hand button press indicated “yes.” In addition, participants’ respiration was monitored and recorded throughout the olfactory paradigm using a chest belt sensor.

### Imaging acquisition

A Siemens 3.0 T system (Magnetom Trio, Siemens Medical Solutions, Erlangen, Germany) with an 8 channel head coil was used to acquire MR images of the entire brain using BOLD sensitive T_2_*-weighted echo planar imaging. The following parameters were used in the sequence: relaxation time (TR) = 2000 ms, echo time (TE) = 30 ms, flip angle (FA) = 90°, field of view (FOV) = 230 × 230 mm^2^, acquisition matrix = 80 × 80, slices = 34, slice thickness = 4 mm, acceleration factor = 2, acquisition time (TA) = 7 min 48 s, and number of repetitions = 234. T_1_-weighted MPRAGE anatomical images were also acquired for overlay for the functional data with the following parameters: TR = 1540 ms, TE = 2.34 ms, FOV = 256 × 256 × 176 mm^3^ , acquisition matrix = 256 x 256 × 176, and TA = 4 min 32 s..

### Data processing

The fMRI data were pre-processed using SPM8 software (Wellcome Trust Centre for Neuroimaging, University College London, UK) with standard parameters, including slice-timing correction, realignment, co-registration, normalization to the Montreal Neurological Institute (MNI) brain template [34], and smoothing (8 × 8 × 8 mm^3^ Gaussian kernel) [35]. The first 5 images were removed to eliminate early transient signal fluctuations [36]. The statistical parametric map (SPM) of each participant was calculated using motion-corrected, normalized functional data after convolving with a canonical hemodynamic response function (family-wise error corrected, p < 0.05, extent threshold = 10). Contrasts were generated for both the odor+visual and visual-only conditions.

This study included analysis of BOLD activity in several ROIs, including the POC, insula, hippocampus, and dlPFC, which we defined *a priori*. The POC consisted of regions that receive direct projections from the olfactory bulb including the piriform cortex, anterior olfactory nucleus, olfactory tubercle, entorhinal cortex, amygdala, and periamygdaloid cortex, and is thus, considered to be one of the earliest sites of central olfactory processing [12]. The insula is also a common site of fMRI activation for both olfactory and gustatory stimulation. In particular, the agranular (anterior) insula, which receives projections from the olfactory tract [37], and the right central insula have been consistently found to be activated in response to olfactory stimulation [38, 39]. Likewise, the hippocampus receives direct input from the entorhinal cortex, which is part of the POC, and has been shown to be involved in odor memory and olfactory-based spatial learning [36, 40-43]. Finally, the dlPFC has been shown to be extensively involved in executive functioning and working memory, possessing a critical network with the hippocampus [44, 45]. Therefore, it was expected that the dlPFC would be activated during this paradigm due to the demand of working memory and associative learning processes. The ROI of the POC was generated by manual segmentation of the T_1_-weighted anatomical images using FMRIB Software Library View (FSLview, Analysis Group, FMRIB, Oxford, UK) [46]. The ROIs of the insula and hippocampus were generated based on the segmented standard brain atlas from AAL (http://www.cyceron.fr/index.php/en/plateforme-en/freeware), while the dlPFC was generated based on the observed activation maps of the fMRI data [47].

Group comparisons with *a priori* masks were performed in SPM8 using a one sample t-test for both the odor+visual and the visual-only conditions. In addition, MarsBaR was used to investigate the BOLD response from all of the previously mentioned ROIs [48]. Each ROI was analyzed for age effects, as well as any relationships between the behavioral olfactory test scores.

Linear regression was also performed to evaluate the effect of age on olfactory function. In order to observe this effect, age was used as a covariate and correlation analyses were performed. In addition, correlation analyses were undertaken using the UPSIT scores to assess the relationship of the scores with the BOLD response of each ROI. Sex differences in olfactory function were also assessed for each ROI for both odor+visual and visual-only conditions by directly comparing the calculated age effect slopes of male and female participants using GraphPad Prism (p < 0.05).

A unique aspect of this study was the use of brain connectivity analysis using euSEM to understand sex differences in olfactory function in terms of effective functional connectivity [49]. Specifically, euSEM was combined with the Group Iterative Multiple Model Estimation technique to evaluate sex differences in causal connections of the optimal olfactory network for this fMRI paradigm [50]. This technique generates a model of effective connectivity, or the influence of one neural system over another, and provides a map of the directional couplings between predefined ROIs [51, 52]. Unlike other effective connectivity modeling techniques, euSEM combines the vector autoregression and standard structural equation modeling to accurately model the influence of specific stimuli on ROI BOLD responses during event-related fMRI designs [53]. In addition, this technique is able to estimate both contemporaneous (at the same time point) and lagged (different time points) connections, making it an optimal method for investigating the dynamics of event-related fMRI data [49, 51, 53]. Specifically in this study, euSEM was used to investigate effective functional connectivity differences between the sexes by directly comparing the presumed olfactory networks for the current event-related design for male and female participants.
